# Chromosomal microarray analyses from 5778 patients with neurodevelopmental disorders and congenital anomalies in Brazil

**DOI:** 10.1038/s41598-022-19274-6

**Published:** 2022-09-07

**Authors:** Ana C. V. Krepischi, Darine Villela, Silvia Souza da Costa, Patricia C. Mazzonetto, Juliana Schauren, Michele P. Migliavacca, Fernanda Milanezi, Juliana G. Santos, Gustavo Guida, Rodrigo Guarischi-Sousa, Gustavo Campana, Fernando Kok, David Schlesinger, Joao Paulo Kitajima, Francine Campagnari, Debora R. Bertola, Angela M. Vianna-Morgante, Peter L. Pearson, Carla Rosenberg

**Affiliations:** 1 The Human Genome and Stem Cell Research Center, Department of Genetics and Evolutionary Biology, Institute of Biosciences, University of São Paulo, Rua do Matão 277, São Paulo, ZIP Code 05508-090 Brazil; 2 Diagnósticos da América S.A., DASA, São Paulo, Brazil; 3 Mendelics, São Paulo, SP Brazil; 4 Instituto da Criança Do Hospital das Clínicas da Faculdade de Medicina da Universidade de São Paulo, São Paulo, Brazil

**Keywords:** Clinical genetics, Cytogenetics

## Abstract

Chromosomal microarray analysis (CMA) has been recommended and practiced routinely since 2010 both in the USA and Europe as the first-tier cytogenetic test for patients with unexplained neurodevelopmental delay/intellectual disability, autism spectrum disorders, and/or multiple congenital anomalies. However, in Brazil, the use of CMA is still limited, due to its high cost and complexity in integrating the results from both the private and public health systems. Although Brazil has one of the world’s largest single-payer public healthcare systems, nearly all patients referred for CMA come from the private sector, resulting in only a small number of CMA studies in Brazilian cohorts. To date, this study is by far the largest Brazilian cohort (n = 5788) studied by CMA and is derived from a joint collaboration formed by the University of São Paulo and three private genetic diagnostic centers to investigate the genetic bases of neurodevelopmental disorders and congenital abnormalities. We identified 2,279 clinically relevant CNVs in 1886 patients, not including the 26 cases of UPD found. Among detected CNVs, the corresponding frequency of each category was 55.6% Pathogenic, 4.4% Likely Pathogenic and 40% VUS. The diagnostic yield, by taking into account Pathogenic, Likely Pathogenic and UPDs, was 19.7%. Since the rational for the classification is mostly based on Mendelian or highly penetrant variants, it was not surprising that a second event was detected in 26% of those cases of predisposition syndromes. Although it is common practice to investigate the inheritance of VUS in most laboratories around the world to determine the inheritance of the variant, our results indicate an extremely low cost–benefit of this approach, and strongly suggest that in cases of a limited budget, investigation of the parents of VUS carriers using CMA should not be prioritized.

## Introduction

Chromosomal microarray analysis (CMA), including array-comparative genomic hybridization (aCGH) and SNP-array, has become the gold standard procedure to detect copy number variations (CNVs) in the clinical setting. Because CMA offers a much higher diagnostic yield (15–20%) than the conventional G-banded karyotype (~ 3%), the test is recommended as the first-tier cytogenetic test for patients with unexplained neurodevelopmental delay/intellectual disability, autism spectrum disorders, or multiple congenital anomalies^1^. It is noteworthy that G-banded karyotyping should be offered only for patients with obvious chromosomal syndromes (e.g., Down syndrome), a family history of chromosomal rearrangement, or multiple miscarriages. However, in Brazil, detection of chromosomal alterations is still performed mainly by karyotyping, due to the high taxation costs of importing microarray material, and relatively cheap technical labour. As a result, the number of CMA studies in cohorts of patients with neurodevelopmental disorders and congenital anomalies is very scarce, and their sample size is typically small (< 500 individuals)^2–6^. Nonetheless, the few previous investigations reported a diagnostic rate ranging from 15 to 22%, similar to that cited in the literature^7–12^.

The clinical interpretation of CMA results can be challenging. Although it is now possible to screen the human genome for CNVs at high resolution, identifying several clinically recognizable syndromes, there are many variants that are rare or only present in a particular individual or family. While some can be confidently predicted to be either pathogenic or benign, in many cases, definitive evidence is missing, leaving us with many variants of uncertain significance (VUS)^13^. Variant classification is often complicated since the criteria applied in the interpretation of a CNV include inheritance, size, type (duplication or deletion), and gene content, with support of multiple database resources for annotation^14,15^. Because the CMA data from the Brazilian population is underrepresented in the literature and public databases, this study was established to provide a collection of genomic data from 5778 patients with various neurodevelopmental disorders; all were genotyped using a high-resolution SNP-array platform. This study is by far the largest Brazilian cohort investigated in diagnostic CMA; by creating this data resource we aimed to establish an overview of all cytogenetic alterations found in clinical CMA, and document the CNVs that are clinically relevant in the diagnosis of neurodevelopmental disorders.

## Material and methods

### Casuistic

The cohort presented here results from a joint collaboration between the Human Genome and Stem Cell Research Center of the Institute of Biosciences, University of São Paulo (IB-USP), and three private diagnostic centers located in the state of São Paulo (DASA, Mendelics, and Deoxi Biotechnology) to provide the largest copy number data from patients investigated in a clinical CMA routine in Brazil. Despite the three diagnostic centers being in the same state in Brazil, patients were referred from all regions of the country. A total of 5778 children underwent CMA, between 2010 and 2020, for presenting a general neurodevelopmental disorder and/or congenital abnormalities without evident cause. All relevant variants are publicly available at the DECIPHER database (https://www.deciphergenomics.org/). This study was approved by the IB-USP Research Ethical Committee, and an informed consent was obtained from the patients’ parents or guardians.

### Chromosomal microarray analysis (CMA): SNP-array

Genomic DNA samples were extracted from peripheral blood cells or saliva following standard procedures. SNP-array experiments were performed using the Illumina Infinium CytoSNP 850 K BeadChip (Illumina, San Diego, USA), except for 810 cases which were carried out using the Affymetrix CytoScan 750 K Array (Affymetrix, Santa Clara, USA). Data were analyzed using either the BlueFuse™ Multi Analysis (Illumina, San Diego, USA) or the Chromosome Analysis Suite—ChAS Software (Affymetrix, Santa Clara, USA). Log2 ratio and B Allele Frequency (BAF) values were plotted along chromosomal coordinates, allowing the detection of both copy number changes and copy neutral regions of homozygosity (ROH).

### Variant analysis and clinical interpretation

Copy number variants were classified for their clinical impact according to the *American College of Medical Genetics* (ACMG) guidelines^15^. The criteria for variant classification were as follow:

- Pathogenic = (1) when the CNVs were more than 4 Mb in length harboring genes, or; (2) overlapped with regions associated with OMIM morbid genes or DECIPHER/ClinGen microdeletion/microduplication syndromes; (3) deleted haploinsufficiency of OMIM genes;
- Likely Pathogenic = when the CNVs (1) were deletions partially affecting haploinsufficiency of OMIM genes; (2) harboring genes and were 1- 4 Mb in length;
- VUS = when the CNVs (1) were duplications containing MIM genes; (2) were deletions, containing recessive MIM genes; or (3) when the segment was larger than 300 kb and harbored genes.

The common variants, i.e., those commonly reported in curated databased (DGV) were disregarded from this study. Chromosomal rearrangements were defined by the presence of more than one large CNV in different or in the same chromosome (e.g.: chromosomes derived from translocations and inversions). In particular, copy neutral ROHs restricted to a single chromosome, known to harbor imprinted regions, were considered pathogenic and likely representing uniparental disomy (UPD). ROH > 10 Mb or at least two ROH > 5 Mb were considered indicative of consanguinity, albeit not Pathogenic per se*.*

### Ethical approval

This study is in accordance with ethical standards established in the Declaration of Helsinki (1964), its subsequent revisions, and Resolution 466/2012 of the Brazilian National Health Council. The Research Ethics Committee of the Institute of Biosciences from the University of São Paulo gave ethical approval for this work (CAAE 80921117.5.0000.5464), and an informed consent was obtained from the patients’ parents or guardians for genetic testing.

## Results

### Diagnostic rate of the cohort

**Figure 1:**
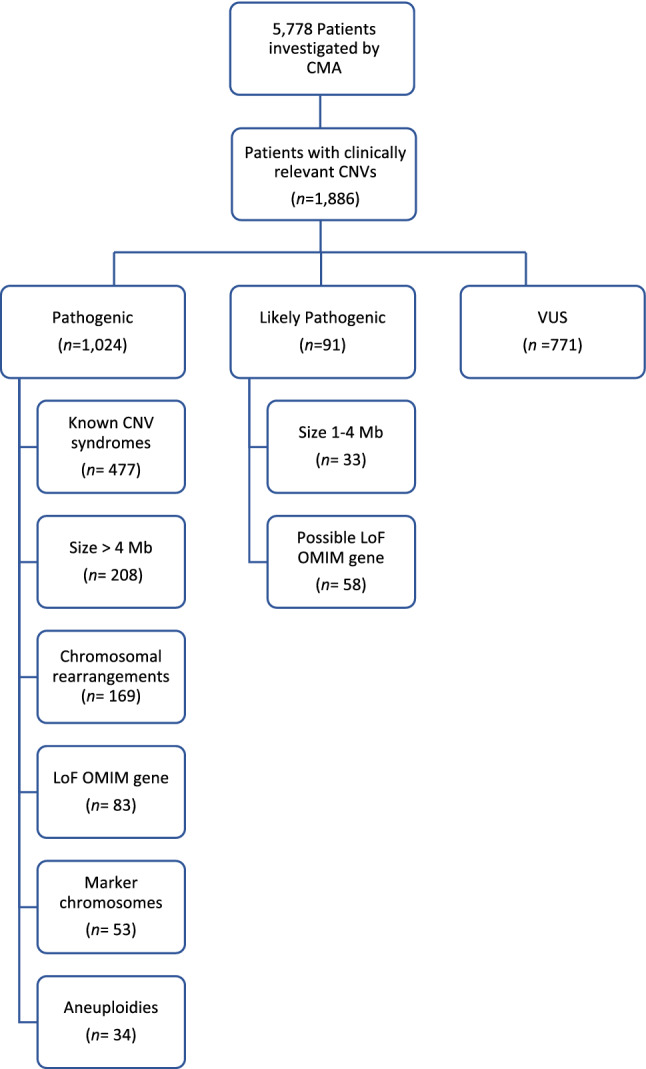
An overview of cases with clinically relevant copy number variations (CNVs) identified in the cohort. The figure shows that, from a total of 5778 patients with neurodevelopmental disorders referred for chromosomal microarray analysis (CMA), 1886 carried clinically relevant CNVs. classified into three main categories: (i) Pathogenic CNVs; (ii) Likely Pathogenic CNVs; and (iii) Variants of Unknown Significance (VUS). The total number of cases corresponding to each category is presented in the diagram. Those individuals with more than one alteration were classified within the most clinically relevant category.

An overview of the number of individuals with clinically relevant CNVs obtained in the cohort of this study is shown in Fig. 1. Out of the 5778 patients with neurodevelopmental disorders or congenital abnormalities investigated, relevant CNVs were detected in 1886 individuals, and were classified in three main categories: (i) Pathogenic (54%); (ii) Likely Pathogenic (5%) and (iii) Variants of Unknown Significance (VUS; 41%). Taking into account just the Pathogenic, Likely Pathogenic and UPD cases, the overall diagnostic yield in our cohort was 19.7%.

**Figure 2:**
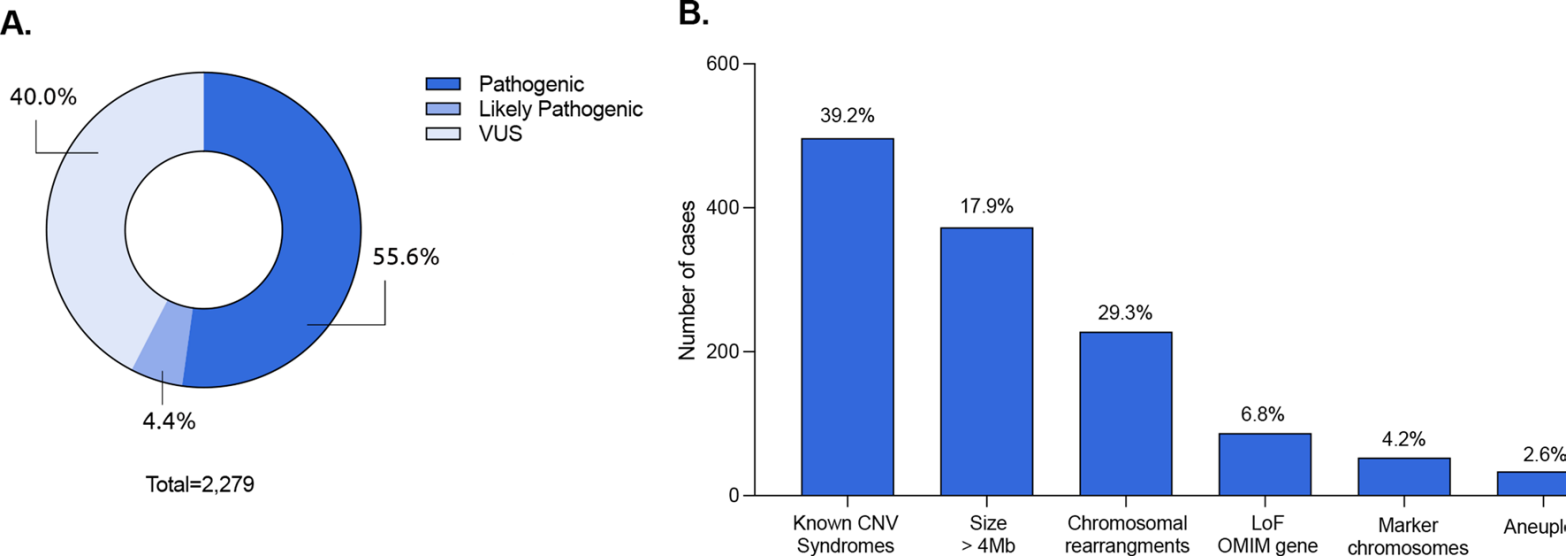
Distribution of the CNVs identified in the cohort. (**A**) It is shown the frequency of variants in each of the three main CNV categories: (i) Pathogenic; (ii) Likely pathogenic and (iii) Variants of Unknown Significance (VUS). (**B**) Distribution of the pathogenic CNVs, displayed by frequency order: (i) known CNV syndromes, (ii) CNV > 4 Mb, (iii) complex rearrangements, (iv) loss-of-function (LoF) MIM gene, (v) marker chromosomes, (vi) aneuploidies.

In these 1886 individuals, a total of 2,270 relevant CNVs, were identified and the corresponding frequency of each category was 55.6% Pathogenic, 4.4% Likely Pathogenic and 40% VUS (Fig. 2A). As expected, Pathogenic CNVs accounted for the largest proportion of diagnostic alterations and are divided into seven clinically relevant classes of variants, as presented in Fig. 2B. The description of all individual Pathogenic, Likely pathogenic CNVs, and VUS can be found in Supplementary Tables 1–3.

### Aneuploidies and marker chromosomes

**Figure 3:**
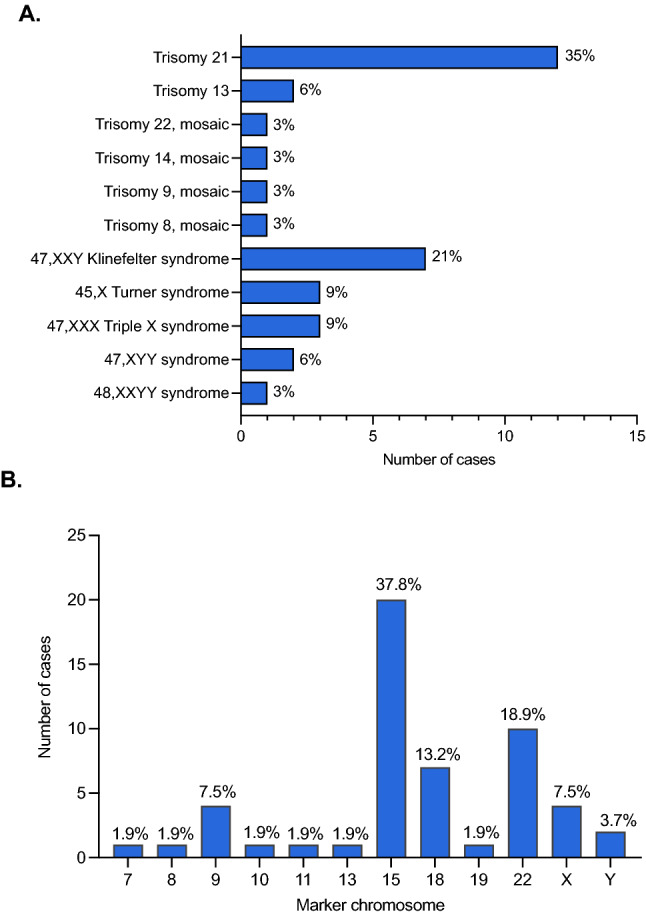
Frequency of aneuploidies and marker chromosomes. (**A**) Sex chromosome aneuploidies (SCA) and autosomal trisomies accounted for a total of 34 cases, in which 16 correspond to SCA and 18 to autosomal trisomies. The histogram shows the frequency of aneuploidies for each chromosome. (**B**) The frequency of the detected 53 marker chromosomes is displayed according to its chromosome origin.

Sex chromosome (SCA) and autosomal aneuploidies accounted for 34 cases (3.3% of the pathogenic cases) in our cohort. Considering them as a separate groups, the proportion was very similar between SCA and autosomal trisomies: 16 and 18 cases, respectively. SCA comprise 47,XXX, 48,XXYY, 47,XYY, 47,XXY, and 45,X; the most frequent being 47,XXY (Klinefelter syndrome), found in 8/34 cases (23.5%). Among the autosomal trisomies, the most common was trisomy 21, found in 12/34 (35.3%), followed by trisomy 13 in 2/34 (5.9%). Excluding the known viable autosomal trisomies, an extra copy of other autosomes was only observed in mosaics, as it was the case with chromosomes 8, 9, 14 and 22. The frequency of each aneuploidy is shown in Fig. 3A. Marker chromosomes were identified in 53 patients (5.2% of the pathogenic cases). We only considered in this category those markers seen in karyotype, and which did not characterize a well-known OMIM syndrome, such as Pallister-Killian, Cat-eye or Emanuel syndromes. Marker chromosome 15 was the most frequent, corresponding to 20/53 (37.8%). Other markers originated from chromosomes 7, 8, 9. 10, 11, 12, 13, 18, 19, 22, X and Y; except for those derived from the sex chromosomes, all were supernumerary marker chromosomes (Fig. 3B).

### Large CNVs and known syndromes

**Figure 4:**
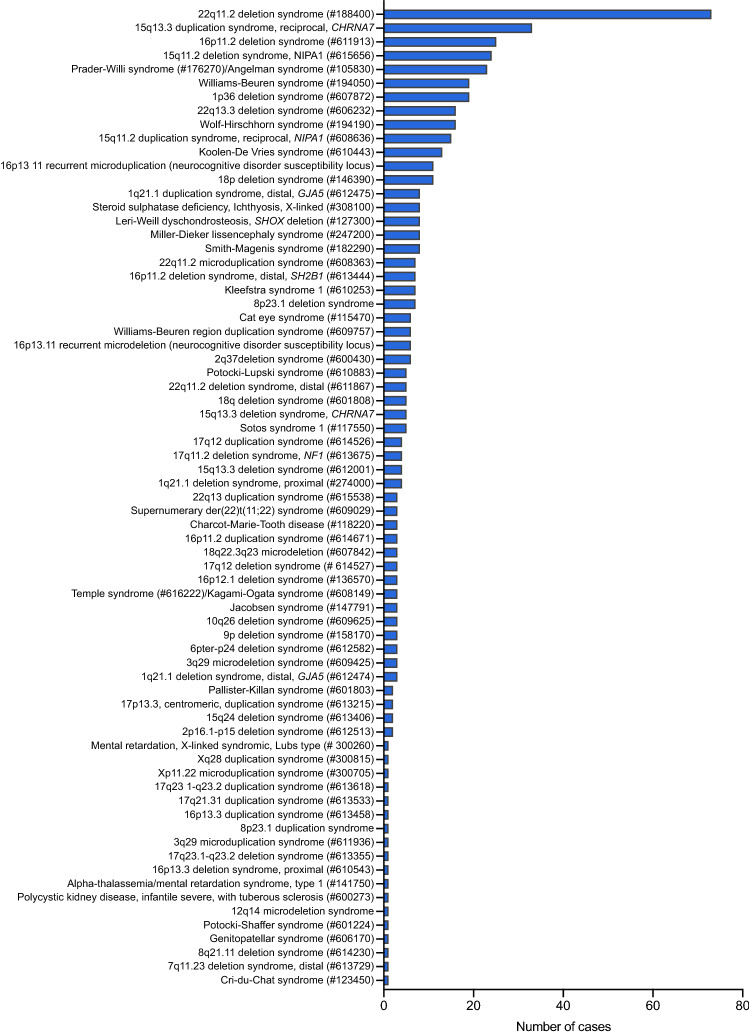
Distribution of microduplication and microdeletion syndromes. The histogram shows the frequency of microduplication and microdeletion syndromes identified in a total of 477 patients with neurodevelopmental disorders. Results are displayed in the descending order of frequency.

**Figure 5:**
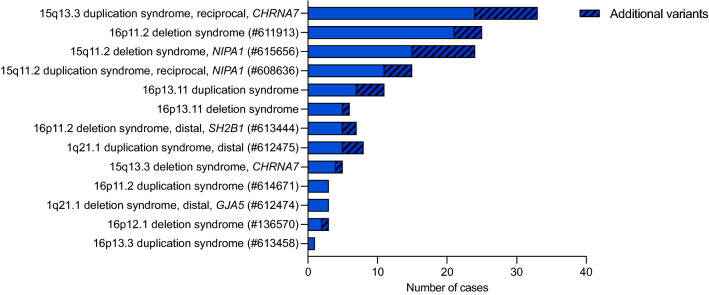
Frequency of syndromes with incomplete penetrance associated with additional variants. The histogram shows the frequency of a secondary CNV associated with a syndrome with incomplete penetrance.

As shown in Fig. 1, we detected isolated large CNVs (> 4 Mb) in 208/1,024 Pathogenic cases (20.3%), and affecting at least two chromosome segments (chromosome rearrangements) in 169/1,024 cases (16.5%). Loss-of-function (LoF) mutations in haploinsufficiency MIM genes accounted for 83 cases (8.1% of the Pathogenic cases). A total of 477 patients (46.6% of the Pathogenic cases) presented 497 CNVs associated with microdeletion or microduplication syndromes (48 deletions and 23 duplications). The five most frequent syndromes were 22q11.2 deletion (14.6%; MIM#188400); 15q13.3 reciprocal duplication encompassing only the *CHRNA7* gene^16^ (6.6%); 16p11.2 deletion (5%, MIM#611913); 15q11.2 deletion (4.8%; MIM#615656); and Prader-Willi/Angelman syndrome (4.6%; MIM#176270/105830, respectively). A detailed listing and the corresponding number of cases and frequencies of each of the 71 clinical entities are shown in Table 1 and Fig. 4. Importantly, among these syndromes, there were 13 known to confer susceptibility to neurodevelopmental disorders, in other words, presented reduced penetrance; such susceptibility CNVs were detected in 144 patients, 38 of which carried additional variants, observed only in autosomes (Fig. 5).

**Table 1:** Frequency of the known copy number variation (CNV) syndromes.

Known CNV syndromes	No. of cases (%)	Cytoband	MIM (#)
**A. Microdeletion syndromes**			
22q11.2 deletion syndrome	73 (14.6%)	22q11.21	188,400
16p11.2 deletion syndrome, 593 kb	25 (5.0%)	16p11.2	611,913
15q11.2 deletion syndrome (*NIPA1*)	24 (4.8%)	15q11.2	615,656
Prader-Willi/Angelman syndrome	23 (4.6%)	15q11.2	176,270/105,830
1p36 deletion syndrome	19 (3.8%)	1p36.2	607,872
Williams-Beuren syndrome	19 (3.8%)	7q11.23	194,050
Wolf-Hirschhorn syndrome	16 (3.2%)	4p16.3	194,190
22q13.3 deletion syndrome	16 (3.2%)	22q13	606,232
Koolen-De Vries syndrome	13 (2.6%)	17q21.31	610,443
18p deletion syndrome	11 (2.2%)	18p	146,390
Smith-Magenis syndrome	8 (1.6%)	17p11.2	182,290
Miller-Dieker lissencephaly syndrome	8 (1.6%)	17p13.3	247,200
Leri-Weill dyschondrosteosis, *SHOX* deletion	8 (1.6%)	Xp22.33	127,300
Steroid sulphatase deficiency/Ichthyosis, X-linked	8 (1.6%)	Xp22.31	308,100
8p23.1 deletion syndrome	7 (1.4%)	8p23.1	https://www.deciphergenomics.org/syndrome/39/overview
Kleefstra syndrome 1	7 (1.4%)	9q34.3	610,253
16p11.2 deletion syndrome, distal, 220 kb – *SH2B1*	7 (1.4%)	16p11.2	613,444
16p13.11 recurrent microdeletion (neurocognitive disorder susceptibility locus)	6 (1.2%)	16p13.1	https://www.deciphergenomics.org/syndrome/79/overview
2q37 deletion syndrome	6 (1.2%)	2q37.2	600,430
Sotos syndrome 1	5 (1.0%)	5q35.3	117,550
18q deletion syndrome	5 (1.0%)	18q	601,808
22q11.2 deletion syndrome, distal	5 (1.0%)	22q11.2	611,867
15q13.3 deletion syndrome (*CHRNA7*) 500 kb	5 (1.0%)	15q13.3	612,001
1q21.1 deletion syndrome, proximal	4 (0.8%)	1q21.1	612,474
15q13.3 deletion syndrome	4 (0.8%)	15q13.3	612,001
17q11.2 deletion syndrome, *NF1*	4 (0.8%)	17q11.2	613,675
1q21.1 deletion syndrome (*GJA5*), distal	3 (0.6%)	1q21.1	612,474
3q29 microdeletion syndrome	3 (0.6%)	3q29	609,425
6pter-p24 deletion syndrome	3 (0.6%)	6p25	612,582
9p deletion syndrome	3 (0.6%)	9p	158,170
10q26 deletion syndrome	3 (0.6%)	10q26	609,625
Jacobsen syndrome	3 (0.6%)	11q23	147,791
Temple syndrome /Kagami-Ogata syndrome	3 (0.6%)	14q32.2	616,222/608,149
17q12 deletion syndrome	3 (0.6%)	17q12	614,527
18q22.3q23 microdeletion	3 (0.6%)	18q22.3q23	607,842
16p12.1 deletion syndrome, 520 kb	3 (0.6%)	16p12.1	136,570
2p16.1-p15 deletion syndrome	2 (0.4%)	2p16.1-p15	612,513
15q24 deletion syndrome	2 (0.4%)	15q24	613,406
Cri-du-Chat syndrome	1 (0.2%)	5p	123,450
7q11.23 deletion syndrome, distal, 1.2 Mb	1 (0.2%)	7q11.23	613,729
8q21.11 deletion syndrome	1 (0.2%)	8q21.11	614,230
Genitopatellar syndrome	1 (0.2%)	10q22.2	606,170
Potocki-Shaffer syndrome	1 (0.2%)	11p11.2	601,224
12q14 microdeletion syndrome	1 (0.2%)	12q14	https://www.deciphergenomics.org/syndrome/76/overview
Polycystic kidney disease, infantile severe, with tuberous sclerosis	1 (0.2%)	16p13.3	600,273
Alpha-thalassemia/mental retardation syndrome, type 1	1 (0.2%)	16p13.3	141,750
16p13.3 deletion syndrome, proximal	1 (0.2%)	16p13.3	610,543
17q23.1-q23.2 deletion syndrome	1 (0.2%)	17q23.1-q23.2	613,355
**B. Microduplication syndromes**			
15q13.3 duplication syndrome, reciprocal (*CHRNA7*) 500 kb	33 (6.6%)	15q13.3	https://dosage.clinicalgenome.org/clingen_gene.cgi?sym=CHRNA7&subject =
15q11.2 duplication syndrome, reciprocal (*NIPA1*)	15 (3.0%)	15q11.2	https://dosage.clinicalgenome.org/clingen_gene.cgi?sym=NIPA1&subject =
16p13.11 recurrent microduplication (neurocognitive disorder susceptibility locus)	11 (2.2%)	16p13.1	https://www.deciphergenomics.org/syndrome/80/overview
1q21.1 duplication syndrome (*GJA5*), distal	8 (1.6%)	1q21.1	612,475
22q11.2 microduplication syndrome	7 (1.4%)	22q11.21	608,363
Williams-Beuren region duplication syndrome	6 (1.2%)	7q11.23	609,757
Cat eye syndrome	6 (1.2%)	22q11	115,470
Potocki-Lupski syndrome	5 (1.0%)	17p11.2	610,883
17q12 duplication syndrome	4 (0.8%)	17q12	614,526
16p11.2 duplication syndrome	3 (0.6%)	16p11.2	614,671
Charcot-Marie-Tooth disease	3 (0.6%)	17p12	118,220
Supernumerary der(22)t(11;22) syndrome	3 (0.6%)	22q11.2	609,029
22q13 duplication syndrome	3 (0.6%)	22q13	615,538
Pallister-Killian syndrome	2 (0.4%)	2p	601,803
17p13.3, centromeric, duplication syndrome	2 (0.4%)	17p13.3	613,215
3q29 microduplication syndrome	1 (0.2%)	3q29	611,936
8p23.1 duplication syndrome	1 (0.2%)	8p23.1	https://www.deciphergenomics.org/syndrome/85/overview
16p13.3 duplication syndrome	1 (0.2%)	16p13.3	613,458
17q21.31 duplication syndrome	1 (0.2%)	17q21.31	613,533
17q23.1-q23.2 duplication syndrome	1 (0.2%)	17q23.1-q23.2	613,618
Xp11.22 microduplication syndrome	1 (0.2%)	Xp11.2	300,705
Xq28 duplication syndrome	1 (0.2%)	Xq28	300,815
Mental retardation, X-linked syndromic, Lubs type, MECP2 duplication	1 (0.2%)	Xq28	300,260
Total	499

### Likely Pathogenic variants and VUS

Lastly, a total of 91 patients carried 99 Likely Pathogenic CNVs, which represent only 4.8% of the individuals carrying CNVs in our cohort (91/1886). The third category of CNVs, the VUS accounted for 908 variants in 771 individuals.

### Uniparental disomy (UPD) and copy neutral regions of homozygosity (ROH)

**Figure 6:**
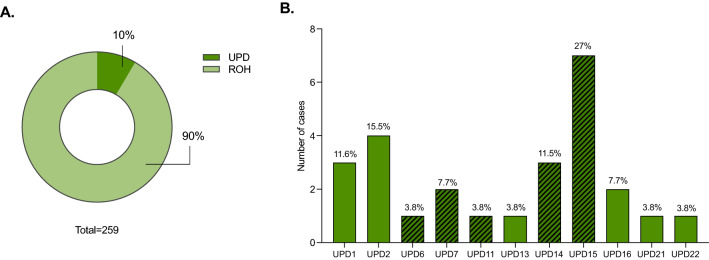
Frequency of copy neutral regions of homozygosity (ROH). (**A**) A total of 259 patients carried ROHs, 26 (10%) corresponding to uniparental disomy (UPD) cases. The remaining ROH in the other 233 patients (90%) were considered associated to different degrees of identity by descendent. (**B**) The histogram shows the frequency of UPDs per chromosome detected in our cohort. The crossed pattern represents pathogenic UPD known to encompass imprinting regions.

Copy neutral ROH were observed in 259 patients and were divided in two categories, according to the supposed origin of the ROH. Twenty-six were large or whole-chromosome ROH block(s) restricted to a single chromosome, classified as UPD; of these UPD cases, 14 were classified as Pathogenic by mapping to imprinted chromosomes 6, 7, 11, 14 and 15. The most common UPD was UPD15 (7/26), followed by UPD14 (3/26) and UPD1 (3/26) (Fig. 6). In the remaining 233 individuals carrying ROHs, the presence of blocks of homozygosity in more than one chromosome was indicative of identity by descent.

## Discussion

In this study, we report the largest Brazilian cohort of patients with neurodevelopmental disorder investigated by CMA. An overall diagnostic yield of 19.7% was determined, a result similar to that found in other studies^8–11^. An extensive study with over 15,000 patients^17^ found a lower frequency of diagnosis (~ 14.2%), but considered only those CNVs above 400 kb. The copy number data presented here was deposited in the DECIPHER database, and clearly demonstrated as previous studies the massive importance of copy number changes in postnatal diagnosis.

CMA has been recommended and practiced routinely in the USA and Europe as the first-tier test for patients with neurodevelopmental disorders and congenital abnormalities since 2010^1^. However, the use of CMA tests is still limited in Brazil due to their high costs. It is relevant to mention that the healthcare system in Brazil is a complex mixture of public and private funding, with governance and ownership agreements. The Brazilian public health sector is one of the world’s largest single payer healthcare systems. In complementation with this scenario there is a significant and large private sector supported with high investment. It is estimated that only ~ 26% of Brazilians have a private health insurance, and it is mainly concentrated in the urban areas of the Southeastern part of the country^18^. Although nearly all patients referred for CMA come from the private sector, the health insurances require that G-banded karyotype be used as the first genetic test. The patients with no structural and/or numerical alterations by karyotyping are subsequently referred for investigation by microarray analysis. In contrast, in the public sector, CMA is not even offered to the patients, since the price established by the government for the total genetic investigation of a patient does not pay even the costs of material for a single CMA. In practice, CMA is provided for few patients at Public Universities or Institutions, when it is linked to specific projects and research grants. However, this situation is not just a Brazilian peculiarity, since in countries with better economic conditions, private laboratories can contribute to a class disparity regarding access to medical analyzes.

Although many patients in our cohort had been previously investigated by G-banded karyotyping, we found 34 cases of aneuploidy, in which trisomy 21 was the most frequent chromosomal disorder encountered. The patients with Down syndrome were referred for CMA for presenting autistic features to search for other CNVs associated with autism spectrum disorders; however, in none of the cases, additional CNVs were detected. It is noteworthy that the presence of autism spectrum disorders in individuals with Down syndrome has been well documented for several years^19,20^; therefore, the analysis through CMA in these cases is puzzling. The second most common aneuploidy detected was the Klinefelter syndrome; also in this case, behavioral disorders were the main reason for CMA referral. It is well documented that behavior problems, including autism, are relatively common in Klinefelter sydnrome^21^. In fact, autistic features may be more common in individuals with sex chromosome aneuploidies than generally believed^22^.

Marker chromosomes from both autosomes and sex chromosomes represented 4.6% of the diagnostic alterations (Pathogenic, Likely Pathogenic, UPD). Except for the X and Y chromosomes, all were supernumerary. The correlation of specific supernumerary marker chromosomes (SMC) with distinct clinical features have been demonstrated for some syndromes; those were classified as syndromes rather than markers, for example i(12p)-(Pallister-Killian) syndrome (MIM#601803), and Cat eye syndromes (MIM#609029 and MIM#115470, respectively)^23^. The only marker that represents a translocation derivative chromosome in our dataset characterize the Emanuel syndrome (MIM#609029), and results from missegregation of the only known recurrent, non-Robertsonian, constitutional translocation in humans [der(22)t(11;22)(q23;q11.2)]. Although it was not possible to obtain cytogenetic characterization of all markers, it is known that an inverted duplicated chromosome 15^24^ is the most common of the heterogeneous group that constitute the supernumerary marker chromosomes.

The microdeletion and microduplication syndromes account for the largest proportion of the diagnosis obtained in our cohort (41.8%). Many of these syndromes harbor genomic hotspots flanked by homologous segmental duplications prone to unequal crossing over, and have high elevated de novo mutation rates, generally with similar CNV sizes^25,26^. In this study, we detected 71 distinct microdeletion/microduplication syndromes in a total of 477 individuals, in which deletions were twice as common than duplications (*n* = 48 deletions vs *n* = 23 duplications). Among the five most frequent syndromes, shown in Fig. 4, four have incomplete penetrance and variable expressivity: 22q11.2 deletion (MIM#188400), 15q13.3 duplication^16^, 16p11.2 deletion (MIM#611913) and 15q11.2 deletion (MIM#615656). Such susceptibility CNVs impose a challenge in genetic counseling since they are present in the normal population but enriched in individuals with various neurodevelopmental disorders. Moreover, these CNVs are often inherited from a normal or mildly affected parent, and they lack phenotypic specificity, being associated with a variety of neuropsychiatric disorders, congenital abnormalities, and variable dysmorphisms.

The 22q11.2 deletion was the most frequent syndrome in our cohort, reflecting the same frequency reported by other large population studies^27^. In particular, the 15q13.3 duplication encompassing the *CHRNA7* gene was previously associated to several neurodevelopmental disorders^17,28,29^. However, overlapping duplications in this genomic region were also documented in many individuals of the general population (~ 0.6%—estimated prevalence of 1:174–186 individuals^30^) and, in almost all cases investigated, patients inherited the duplication from clinically normal parents. The high frequency of duplications of this segment in the general population, together with the lack of enrichment in clinical cohorts^31,32^, indicate that, if this variant has any clinical impact, the penetrance would be very low.

The recurrent 15q11.2 deletion (BP1-BP2), which includes the *CYFIP1*, *NIPA1*, *NIPA2*, *TUBGCP5* genes, is consistently associated with neurocognitive function. Jonch et al. (2019)^33^ performed a comprehensive meta-analysis on individuals with 15q11.2 deletions, comparing data across 20 studies. The case–control study using their clinical cohort compared to controls in the UK Biobank cohort showed enrichment of the deletion in the patient population. Nonetheless, the reciprocal duplication of the 15q11.2 region has refuted clinical significance^31,34^. Duplications of this region are common in the general population and the majority of case–control studies have observed a lack of enrichment in the clinical population. Recent studies of duplication carriers identified through cohort studies in the general population have also shown that carrier individuals perform similarly to non-carrier controls on neurocognitive tests. Therefore, duplication of this segment is considered unlikely dosage sensitivity. Indeed, 15q11.2 and 15q13.3 restricted duplications were historically classified as pathogenic, and the data presented reflects a retrospective analysis; currently, are classified as benign in light of recent evidence, and in accordance with databases such as DECIPHER and ClinGen.

The estimated frequency of the 16p11.2 deletion syndrome is about 1–5/10,000 in the general population. Research based on the ClinGen database suggests that 16p11.2 deletions are the second most common microdeletion, occurring in one of every 235 individuals tested with intellectual and developmental disabilities. Interestingly, this deletion was identified in nearly 1% of individuals with autism^35–37^. Nonetheless, the phenotypic spectrum associated with this deletion is much wider and includes delays in speech or motor development, language impairment, low muscle tone, hypo- or hyperreflexia, a tendency towards obesity, short stature, and several facial dysmorphisms. The syndrome classically involves a heterozygous microdeletion of ~ 600 kb, containing 29 protein coding genes; although the majority of cases reported are de novo, the deletion is inherited in an autosomal dominant pattern in 20% of the cases; an equal sex ratio has been reported^38^.

Considering all evidence from association studies about the susceptibility CNVs for neurodevelopmental disorders, the general consensus is that there must be additional modifiers that influence the expression of these variants. A “two-hit”, or second site, model has been suggested for several syndromes^39,40^. Notably, the vast majority of the syndromes with incomplete penetrance, as shown in Fig. 5 from our cohort, present an additional CNV in part of the patients, mostly classified as VUS. Girirajan et al. (2010) demonstrated that 25% of the affected individuals with a microdeletion on chromosome 16p12.1 carried additional large CNVs ^40^. This data supports an oligogenic basis, in which the compound effect of a relatively small number of rare variants of large effect contributes to the heterogeneity of genomic disorders. The authors also identified other known genomic disorders, each defined by a specific CNV, in which the affected children were more likely to carry multiple copy number variants than controls^39^. Overall, a second CNV hit was identified in 10% of their cases. We found that patients with a CNV known to present incomplete penetrance (Fig. 5) carried a CNV second-hit in 26.4% of the cases. It is assumed that part of the second-hits would be only ascertained by sequencing, but SNV/indel second-hits were not investigated in this study. It is relevant to mention that investigation of the parents is mandatory in cases of those susceptibility CNVs for proper genetic counselling^15^. However, in our study we were unable to obtain information about inheritance pattern for all cases because the patients and parents were not necessarily investigated at the same centers or in the same period of time.

In cases where the patient present one or more VUS, the recommendation is to investigate the parents to determine whether the CNV has been inherited or represents a de novo mutation^15^. While the latter may lead to a reclassification of the VUS as pathogenic or likely pathogenic, the inherited variants remain classified as VUS. In our cohort, we have information on segregation in a minority of the cases: among 126 patients who presented an autosomal VUS and segregation was tested, we found that 125 of the variants were inherited. It is important to remember that, regardless of being inherited, these VUS may still contribute to the patients’ phenotype, and must be reported. While it is undeniable that the resulting 0.8% de novo alterations have some impact in diagnosis and genetic counselling, the healthcare context should be considered in the decision to test the parents. In the present cohort, we performed 252 CMA tests in parents, but were able to reclassify the VUS in a single case. For all remaining 125 cases, the parental tests did not add any useful information. When resources are limited, such as in Brazil and in many other countries, we obtain a better cost–benefit testing other 252 patients instead of investigating VUS segregation. Therefore, we would not recommend testing segregation of VUS using CMA in the public healthcare in Brazil or in other developing countries in the current situation. Nonetheless, segregation analysis can be performed with cheaper techniques such as real time PCR.

Importantly, with the incorporation of many robust SNP-array platforms in the clinical routine, many studies have identified large ROH in patients with a wide variety of clinical features^41–44^. Depending on chromosomal distribution and cumulative extent, it may either indicate UPD or parental consanguinity^45^. When ROH > 10 Mb are detected in a single chromosome, the first possibility to be considered is UPD; this event arises as a consequence of a trisomy rescue, which may have further implications, such as the presence of an undetected trisomic cell line. In cases of chromosomes subject to imprinting, the presence of two copies of the same chromosome inherited from only one of the parents is considered as pathogenic per se. UPD in non-imprinted chromosomes still increases the probability of deleterious mutations in homozygosity^46^ Contrarily, the presence of many ROH throughout the genome is an indication of consanguinity, and the chance of inheritance of recessive monogenic disorders increases with the degree of relatedness. It has been demonstrated that the occurrence of multiple congenital anomalies and other significant clinical problems is higher among children of first cousins (4.4%) and second cousins (3.6%), compared to unrelated parents^47^. The rate of consanguinity in the different regions of Brazil is very heterogeneous and estimates are scarce. A recent paper indicates that some degree of inbreeding may be present in 26.5% of patients with developmental disorders of the South of Brazil^2^. However, when clinically more relevant kinship of 1st–5th degree is considered, they find consanguinity in ~ 8.5% of the cases. In our cohort, only 233 out of the 259 cases of ROH were interpreted as the result of consanguinity, reflecting a lower frequency of consanguinity of 4.0% (233/5778).

In summary, we reported copy number data from patients with neurodevelopmental disorders and congenital anomalies in the largest Brazilian cohort investigated by CMA reported so far. These data, available in the DECIPHER database, can be used as a valuable resource for other genomic studies.

## Supplementary Information

Supplementary Information 1.

Supplementary Information 2.

Supplementary Information 3.

## Data Availability

The datasets generated and/or analyzed during the current study are available in the DECIPHER database (https://www.deciphergenomics.org/). The reference number that corresponds to the DECIPHER patients ID are presented on the Supplementary Tables. All genomic coordinates from the variant data that support the findings of this study are available on the Supplementary Tables. Raw data supporting the findings of this study are available on request from the corresponding author—C.R. The raw data are not publicly available due to patients privacy/consent restriction.
